# The enigmatic nature of altruism in organ transplantation: a cross-cultural study of transplant physicians' views on altruism

**DOI:** 10.1186/1756-0500-3-216

**Published:** 2010-07-30

**Authors:** Marie-Chantal Fortin, Marianne Dion-Labrie, Marie-Josée Hébert, Hubert Doucet

**Affiliations:** 1 Nephrology and Transplantation Division, Centre Hospitalier de l'Université de Montréal, 1560 Sherbrooke East Street, Montreal, Quebec, H2L 4M1, Canada; 2 Bioethics Department, Université de Montréal, P.O. Box 6128, Downtown Station, Montreal, Quebec, H3C 3J7, Canada

## Abstract

**Background:**

Although altruism is a key principle in our current organ donation and transplantation system, the meanings and implications of the term have been widely debated. Recently, a new type of living organ donation--anonymous and non-directed, also called living altruistic donation (LAD)--has brought the issue into sharper focus. Transplant physicians' views on altruism might influence their attitudes and actions toward living altruistic donors. This study aimed to explore such views among transplant physicians in France and Quebec.

**Findings:**

A total of 27 French and 19 Quebec transplant physicians participated in individual, semi-structured interviews between October 2004 and December 2005. The majority of these participants associated altruism with gratuitousness and saw altruistic acts as multiple and varied, ranging from showing consideration to saving a person's life.

**Conclusions:**

The transplant physicians' discourses on altruism were quite diverse, leading us to question the relevance of the concept in organ transplantation and the appropriateness of the term "living altruistic donation."

## Background

Altruism and gift-giving have been an integral part of organ transplantation from the outset: the gift of science to humanity; grieving family members who offer to donate the organ of a deceased loved one; recipients who agree to participate in research. Although organ transplantation has become a routine procedure in the past decade, altruism remains a key principle. Indeed, many organ procurement organizations (OPOs) and medical associations such as United Network for Organ Sharing (UNOS) and the American Society of Transplantation (AST) explicitly state that organ donation and transplantation should be based on altruism [1-4]. The World Medical Association (WMA), in its "Statement on Human Organ Donation and Transplantation," indicates that "Payment for organs for donation and transplantation must be prohibited. A financial incentive compromises the voluntariness of the choice and the altruistic basis for organ donation" [5].

As this citation suggests, altruism, in the context of organ donation, is often narrowly defined as an absence of monetary exchange and commercialization. The concept of altruism has been studied and debated since the very beginnings of organ transplantation. In the case of living organ donation, many of these studies have examined living organ donors' motivations and psychosocial profiles [6,7]. Over the past decade, altruism has again come to the fore with the emergence of the living altruistic donor (LAD), also known as the "Good Samaritan" donor, live unrelated donor, or living anonymous donor. The transplant community feared this new type of living organ donation would open the door to a commercial trade in organs, and that donors in such instances were suffering from some kind of psychiatric pathology [8,9]. Some transplant community members were sceptical about donors' motives, since wanting to donate a kidney to a stranger appears to run counter to self-interest [10]. Although living altruistic donation has been hotly debated in recent years, it has become more widely accepted [11]. One might question the significance of altruism in this context. Is the concept not superfluous or redundant? Is living organ donation not necessarily altruistic, whoever the recipient may be? Does qualifying this type of organ donation as altruistic involve taking a stand on the premises and meaning of altruism?

Healy has shown that altruistic practices are embedded in socio-organizational structures [12,13]. Transplant physicians play a role in these structures. They are members of scientific organizations which inform and educate decision-makers on transplantation issues and help develop policies and regulations [14]. They also sit on the committees and boards of OPOs and associations such as UNOS. Physicians' views and discourses on altruism could therefore potentially influence guidelines on issues such as acceptable motives for living altruistic donation, solicitation of organ donors on the Internet, the organ trade, and so on. Other than a recent article on intensivists' and transplant coordinators' perceptions of the gift relationship in organ donation contexts, there have been no empirical studies on transplant physicians' perspectives on altruism [15]. In order to gain an in-depth understanding of these perspectives, we chose to compare samples from France and Quebec. Both contexts share cultural characteristics such as language, have socialized healthcare systems, and have similar rates of deceased donation. However, they differ in terms of their laws and transplantation practices. In Quebec, there is currently no LAD program, but the issue is the subject of heated debate within the transplant community. In France, LAD is strictly prohibited [16].

## Methods

In this study, we adopted an empirical ethics approach, allowing the facts gathered to inform normative considerations (the "is" contributing to the "ought") [17-19]. This study was part of a larger project aimed at exploring transplant physicians' views on LAD [20-22]. Using a qualitative methodology, we gathered data through semi-directed interviews. In each interview, respondents were asked to provide a general definition of altruism (not related to organ donation) as well as examples of behaviours they considered altruistic.

The study was conducted between October 2004 and December 2005. Respondents in both settings were nephrologists or surgeons involved in the field of renal transplantation. In Quebec, the participants were sampled purposively. A total of 22 physicians (nephrologists and transplant surgeons) from all seven hospitals (adult and pediatric) conducting renal transplantation were contacted; 19 agreed to participate. In France, the transplant directors of seven hospitals located in five cities were contacted (living organ donation rates in these hospitals ranged from 3% to 15%) [5]. Once the transplant directors had agreed to participate, a snowball sampling technique was used. A total of 29 transplant physicians working in different cities were contacted; 27 agreed to participate. The characteristics of the respondents are summarized in Table 1. The number of French and Quebec participants was sufficient to achieve data saturation in both samples [23]. The research ethics committee at Université de Montréal approved the study and all participants gave their informed consent.

**Table 1:** Characteristics of Respondents

	French Physicians N = 27	Quebec Physicians N = 19
**Medical specialty**		
Nephrologist	21	11
Surgeon	6	8
**Gender**		
Male	23	11
Female	4	8
**Milieus**		
Pediatric	9	3
Adult	17	13
Both	1	3
**Age (years)**		
Average	51	48
Range	29-68	33-67
**Years of practice**		
Average	21	17
Range	1-38	2-37

**Open to the idea of living altruistic donation**	14 (52%)	16 (84%)

Interview transcripts were analyzed using the content and thematic analysis method described by Miles and Huberman [24]. The computer software N'Vivo (version 2.0) was used for the qualitative analysis. An independent researcher coded 10% of the raw data, and the rate of coding agreement was subsequently assessed (80%).

## Results

### Definition of altruism

A fundamental characteristic of altruism noted by most of the respondents was gratuitousness (selfless, uncalculated acts of kindness). The imperative of gratuitousness also emerged in the transplant physicians' opposition to any form of payment for organs. Donors should not intend to benefit from the donation and should not expect any kind of reward. However, four of the physicians in France and one in Quebec felt that gratuitousness--and, by extension, altruism--were impossible. Rather, these physicians believed in psychological egoism (i.e., individuals act out of self-interest even when they collaborate with others) [25]. In other words, no act is unmotivated and gratuitous--people always seek to maximize their well-being. One of the French nephrologists remarked that there is always an expectation of personal benefit in every altruistic action. Thus "a generous person is inevitably suspect." It is interesting to note that out of the five transplant physicians who subscribed to the theory of psychological egoism, the Quebec physician was open to LAD, whereas his four French colleagues expressed reservations or were adamantly opposed to this type of donation.

Related to gratuitousness is the pleasure associated with giving or with an altruistic act. Opinions were also divided as to whether this pleasure may be reconciled with altruism or whether it constitutes a reward or an incentive. For some respondents, pleasure makes the act of giving egoistic:

"I would say that an altruistic person is not altruistic, but egoistic .... This person derives pleasure from donating; therefore he is not so generous, because he benefits from donating." (Quebec physician)

Other respondents acknowledged that the pleasure associated with giving might be an acceptable reward for the donor (i.e., did not contradict the principle of altruism) if it was not expected and was not the key motivating factor.

A second feature of altruism described by many of the physicians was its outward or other-focused nature. By definition, an altruistic gesture is directed toward, and involves helping, others. One respondent remarked on the lack of altruism in our society, referring to the many seniors who died during the 2003 heat wave in France, partly as a result of family members' and neighbours' indifference or neglect.

Thirdly, for many respondents, altruism involved gift-giving or charity. In the words of one French physician, "[Altruism] is, above all, a gift--an impressive show of generosity."

A few respondents said that to be considered altruistic, an act should involve some risk or sacrifice on the part of the giver. Altruistic acts should not be easy and are therefore not common:

"Essentially, altruism means giving a part of ourselves, not giving $50 to an organization. Anyone can do that." (Quebec physician)

" ... [to be altruistic] is to be prepared to take risks for others." (Quebec physician)

Taking risks does not mean going so far as to risk one's life. Many physicians found the idea of dying to save someone else--for example, a person who is unable to swim trying to stop someone from drowning--to be unacceptable.

"There is always a cost associated with altruism, but this cost shouldn't be too great. It shouldn't involve sacrificing your life." (French physician)

Also mentioned with regard to altruistic acts was their voluntary and beneficial nature. Finally, one French transplant physician associated altruism with love. For him, the degree of war and conflict in the world proves that altruism does not exist.

### Types of altruistic actions

During the interviews, respondents were asked to list altruistic actions. At one end of the spectrum were acts of common courtesy such as giving up one's seat on the bus; at the other were heroic acts like rescuing someone. In between were actions such as donating money or goods to a charity; volunteer work; deceased organ donation; the donation of blood, sperm, eggs and tissue; humanitarian aid (e.g., Doctors without Borders, the Red Cross); and living organ donation. However, not all respondents considered living organ donation to be altruistic under all circumstances. Wanting to donate one's heart while still alive, or donating a portion of one's lung or liver would not qualify as altruistic intentions or acts because they would involve sacrificing or risking one's life in order to help another person. Respondents mentioned three other situations they did not consider altruistic: helping someone while harming another person, dying for a cause, and neglecting loved ones in order to save strangers. There was a strong sense that charity begins at home.

## Discussion

In his seminal essay on gift exchange in traditional societies, Marcel Mauss argued that gift-giving is governed by three obligations: to give, to receive and to repay. The paradox of a gift exchange, according to Mauss, is that it is free but obligatory. To refuse to participate in a gift exchange is to refuse the other and create a rupture that could lead to war. Gift-giving serves to forge ties and alliances among groups and therefore has a strong moral component [26]. Renée C. Fox and Judith Swazey have shown how Mauss' three obligations apply to organ transplantation. For instance, in the case of living organ donation, family members feel compelled to offer to donate an organ to help their loved one recover his or her good health. The recipient also feels obliged to accept the offer--to do otherwise would be to reject the giver or refuse the relationship. Finally, the obligation of repayment can be problematic with a gift as valuable as an organ. Fox and Swazey describe the "tyranny of gift," a situation that occurs when a strong creditor-debtor relationship between donor and recipient is created [27]. In modern societies, the gift may be repaid over time or to people other than the donor [28]. Sociologist Jacques T. Godbout has shown how gifts are commonly given to strangers. Individuals who have received a lot from their family and close relatives may choose to donate to strangers and other members of their community. In fact, according to Godbout, what drives individuals to donate and reciprocate is the fact that life begins with a gift: a mother giving birth to a child [29].

These scholarly works are focused on gift exchange rather than altruism. However, for many, the two are closely linked. This was certainly the case in this study, where many respondents viewed altruism as equivalent to gift-giving. In a recent article, Shaw has shown that for New Zealand intensivists and transplant coordinators, gift-giving was also linked with altruism [15].

A common denominator in the definitions of altruism provided by the transplant physicians in our study was gratuitousness. Some respondents (mainly the French transplant physicians) felt that the pleasure experienced by donors ruled out the possibility of altruism, since the latter implies an absence of reward or reciprocity. This view coincides with Derrida's view on gift-giving. For this French philosopher, to give means to expect absolutely nothing in return from the recipient. The latter should not acknowledge receiving anything and the donor should not recognize his or her gesture of generosity, as this will lead to feelings of gratification [30]. Is this view a myth or a reality? The French transplant physicians' views on altruism could also be linked to a general suspicion regarding living organ donation. Unlike in Quebec, LAD is strictly prohibited in France. Only spouses or genetically-related persons can be living organ donors. Moreover, potential living organ donors who fulfil the legal requirements have to meet with an external committee to ensure that there is no coercion and that they are well-informed and are consenting freely [16]. A recent study on the perspectives of French transplant physicians shows that they view themselves as morally responsible for the donation--in other words, they feel duty-bound to ensure there is nothing motivating the donation other than generosity and solidarity [31].

It is interesting to consider the relationship between altruism, gratuitousness and gift-giving in light of the cited works by Mauss, Godbout, and Fox and Swazey. A purist stance implies that altruism involves both intent and consequences. How can a gratuitous view of altruism, which excludes any form of return, be reconciled with gift-giving? As seen previously, reciprocity is an integral part of gift-giving. The unclear relationship between gratuitousness, altruism and gift-giving could translate into misunderstandings or uneasiness on the part of transplant physicians. What is the cause of this discomfort? Here, we can only speculate. Are transplant physicians uncomfortable about the thing given, namely an organ? Is it the fact that transplant physicians and institutions are the initial recipients of the gift of an organ? Is it because we use an ethics of gift exchange, generosity and altruism to justify organ procurement and transplantation, and to downplay the associated elements of sacrifice, mutilation and risk of death? Does Western capitalism make us forget that altruism is possible? Further studies are needed to explore these issues.

## Conclusion

This exploratory study describes views on altruism offered by a sample of French and Quebec transplant physicians. These views are embedded in social, cultural, legal and political contexts, and cannot be extrapolated beyond these settings. Our qualitative methodology allowed us to offer some explanations as to why LAD is not widely performed in France and Quebec. The interview data also shed light on how transplant physicians, in their close and daily involvement with organ donation, see altruism. Their perspectives and discourses on the subject help us better understand their assessments, judgments and expectations regarding living organ donors, which subsequently inform the ethical guidelines adopted by medical or transplant communities. Of course, it is important to bear in mind that these views might differ from those of other transplant stakeholders, such as patients on the waiting list, potential donors and the general public. Further qualitative and quantitative studies are needed to explore these views and their bearing on policies and practices.

Altruism plays a central role in transplantation and the promotion of organ donation. However, the concept has multiple meanings for transplant physicians. Our data lead us to question whether altruism is overly simplistic, confused or even useful in the field of organ transplantation. Our study has shown how transplant physicians are uneasy with notions of altruism and gift-giving. Is LAD a misnomer? Shaw has shown that the gift metaphor is useful for the public promotion of organ donation but ambiguous for professionals [15]. Some might argue that it would be more accurate and relevant to speak of generosity, benevolence, empathy, solidarity and love for others. However, are these concepts really more straightforward and less ambiguous? All are related to gift-giving--a complex and enigmatic practice which is nonetheless a fundamental part of human existence.

## Competing interests

In 2006, Marie-Chantal Fortin received an educational grant from Roche Canada to lead a workshop titled "Ethical and Juridical Challenges in the Face of the Organ Shortage" at the World Medical Congress held in Toulouse, France, in August 2006.

## Authors' contributions

MCF designed the study, conducted the interviews, analyzed the data and drafted the manuscript. MDL participated in the data analysis and independently coded 10% of the raw data. She also provided input on the draft manuscript. MJH participated in the review of the manuscript. HD supervised the design and the completion of the study. He helped review the draft manuscript. All authors read and approved the final manuscript.
